# Mesenchymal stromal cell-derived extracellular vesicles reduce lung inflammation and damage in nonclinical acute lung injury: Implications for COVID-19

**DOI:** 10.1371/journal.pone.0259732

**Published:** 2021-11-15

**Authors:** Caryn Cloer, Laila Roudsari, Lauren Rochelle, Timothy Petrie, Michaela Welch, Joseph Charest, Kelly Tan, Li Fugang, Thomas Petersen, Roger Ilagan, Sarah Hogan

**Affiliations:** 1 Department of Regenerative Medicine, United Therapeutics Corporation, Durham, North Carolina, United States of America; 2 Draper, Cambridge, Massachusetts, United States of America; 3 HD Biosciences, Shanghai, China; University of Pittsburgh, UNITED STATES

## Abstract

Mesenchymal stem cell derived extracellular vesicles (MSC-EVs) are bioactive particles that evoke beneficial responses in recipient cells. We identified a role for MSC-EV in immune modulation and cellular salvage in a model of SARS-CoV-2 induced acute lung injury (ALI) using pulmonary epithelial cells and exposure to cytokines or the SARS-CoV-2 receptor binding domain (RBD). Whereas RBD or cytokine exposure caused a pro-inflammatory cellular environment and injurious signaling, impairing alveolar-capillary barrier function, and inducing cell death, MSC-EVs reduced inflammation and reestablished target cell health. Importantly, MSC-EV treatment increased active ACE2 surface protein compared to RBD injury, identifying a previously unknown role for MSC-EV treatment in COVID-19 signaling and pathogenesis. The beneficial effect of MSC-EV treatment was confirmed in an LPS-induced rat model of ALI wherein MSC-EVs reduced pro-inflammatory cytokine secretion and respiratory dysfunction associated with disease. MSC-EV administration was dose-responsive, demonstrating a large effective dose range for clinical translation. These data provide direct evidence of an MSC-EV-mediated improvement in ALI and contribute new insights into the therapeutic potential of MSC-EVs in COVID-19 or similar pathologies of respiratory distress.

## Introduction

A highly infectious disease caused by severe acute respiratory syndrome β-coronavirus 2 (SARS-CoV-2), COVID-19 is often complicated by respiratory failure, acute lung injury (ALI) and its severe form, acute respiratory distress syndrome (ARDS), which contribute significantly to associated morbidity and mortality. Recent studies suggest that among the patients who succumbed to COVID-19, over 90% had confirmed ALI and/or ARDS [1, 2]. Despite the advances in symptom management and major improvements in our understanding of the disease, there remains an urgent need for advanced therapeutics to treat ALI.

Pulmonary elevation of pro-inflammatory cytokines are a hallmark of ALI, intensified in patients with SARS-CoV-2 and correlated to poor clinical prognosis [3]. Local cytokine over-production and sustained systemic inflammation drive alveolar-capillary membrane damage, pulmonary edema, and hypoxemia [3]. Therefore, strategies that modulate the inflammatory cascade may benefit acutely ill patients with ALI due to SARS-CoV-2. Treatments that have anti-inflammatory effects have been identified as a key priority in SARS-CoV-2 research [4]. Administration of mesenchymal stem cells (MSC) has been one such avenue with promising preliminary clinical outcomes [5, 6]. However, stem cell transplants are often complicated by concerns over cellular aggregation, uncontrolled cell division, and graft-versus-host complications [7]. Subsequent studies demonstrated that paracrine signaling in the form of MSC-derived extracellular vesicles (MSC-EVs) could harness the benefits of stem cell therapy without the drawbacks associated with cellular transplantation [8, 9]. Indeed, promising work has described an MSC-EV-mediated reduction in pulmonary inflammation and edema in models of lung injury [10, 11]. Importantly, MSC-EVs have an anti-inflammatory and anti-viral impact in preclinical models of influenza infection and endotoxin exposure [9, 12]. These data and the critical need for COVID-19 treatments have led to the initiation of several MSC-EV clinical trials to treat critically ill patients with COVID-19 ALI [13]. Despite these promising studies, preclinical efficacy data for the use of MSC-EVs is limited. Further, the impact of MSC-EV on COVID-19 signaling is poorly understood.

Herein, we sought to determine the impact of MSC-EVs on COVID-19 induced ALI. To that end, we identified a treatment effect of MSC-EVs on alveolar-capillary damage and inflammation. We establish an important role for MSC-EVs in reducing damaging cellular signaling due to SARS-CoV-2. Because rodents lack an equivalent ACE2 receptor to human, non-clinical models of SARS-CoV-2 viral infection are limited [14]. Thus, we utilized a rat model of LPS exposure to model clinical characteristics of ALI distinct from ACE2 regulation and demonstrate the utility of MSC-EV in preventing ALI progression and in symptom management. We reveal a dose-responsive reduction in lung inflammation and injury following LPS-induced tissue damage. Taken together, these data are consistent with a role for MSC-EVs in the treatment of COVID-19 ALI.

## Materials and methods

### Extracellular vesicles

MSC-EVs were prepared as described previously [15]. Briefly, primary human bone marrow-derived mesenchymal stromal cells (MSCs; RoosterBio) were grown in KT-020 growth media (RoosterBio) until 100% confluent. Cells were then washed twice with PBS and incubated in serum-free, protein-free basal media for 48 h. Conditioned media was collected, filtered (0.2 μm filter), and adjusted to 10 mM EDTA and 25 mM HEPES. Conditioned media was concentrated by tangential flow filtration (Sartorius), and MSC-EVs were purified using a size exclusion column packed with Sepharose CL-2B resin (GE Healthcare).

#### Nanoparticle tracking

Evaluated as described [15] by Particle Technology Labs. Briefly, MSC-EVs were diluted to 6.5X and injected into the Malvern NanoSight, which then generates video of nanoparticles moving under Brownian motion in liquid while illuminated by a laser. Videos are then analyzed using the Malvern NanoSight NTA analysis software to determine particle size and concentration.

#### Tetraspanins

Antibody microarray bound MSC-EVs were detected at Nanoview Biosciences using reflectance imaging as previously described [16]. Briefly, 50 μL MSC-EV was mixed with 0.5 μL 1% tween-20 and loaded onto ExoView chips containing immobilized CD63, CD81, CD9 antibodies or corresponding isotype controls. Sample incubation was run for 16 hours at room temperature. Chips were then washed in isotonic buffer containing mild detergent, then deionized water, dried, and analyzed using ExoView R100 instrument and NanoViewer 2.9.1 software. Tetraspanin concentration was additionally evaluated at MesoScale Discovery using an electrochemiluminescent multiplexed immunoassay platform and antibodies labeled with an ECL emitting tag.

MSC-EVs were loaded neat into assay wells containing antibodies against CD63, CD81, CD9, or appropriate IgG1 negative controls (MSD cat# S01CD-1). MSC-EV raw ECL signals were normalized to antibody control.

#### Western blot

MSC-EVs lysates were prepared using 30X ethanol precipitation into RIPA lysis buffer. Lysates were volume loaded and separated on a 12% Bolt Bis-Tris Gel and probed using Abcam antibodies against flotillin-1 (ab133497 at 1:10,000 dilution), Annexin-2 (ab41803 at 1:1000 dilution), Syntenin-1 (ab19903 at 1:1000 dilution), MHC-I (ab110645 at 1:1000 dilution), MHC-II (157210 at 1:10000 dilution), and *Calreticulin* (ab92516 at 1:1000 dilution).

#### Immunogold imaging

MSC-EVs were prepared using Cryo-transmission electron microscopy and specific gold labeling against CD63 and phosphatidylserine as previously described at Exo-Analysis [17].

#### Atomic force microscopy

MSC-EVs were mounted directly on poly-lysine treated mica and imaged using atomic force microscopy at SurfaceChar.

#### Phospholipid analysis

MSC-EV were evaluated for phospholipid content using HPLC-MS analysis at Alera Labs.

### Cell culture

#### THP1 cells

Human THP1 monocytes (ATCC cat# TIB-202, RRID: CVCL_0006) were cultured in RPMI with 0.5% FBS, 0.05 mM β-ME and treated with 0.2 ng/μL lipid A monophosphoryl from *Salmonella*, *minnesota* R595 (LPS, Lisa Biological labs #401) for 24 hours prior to media collection for further analysis.

#### Calu3 cells

The human airway epithelial cell line Calu-3 (ATCC HTB-55, RRID: CVCL_0609) was cultured in antibiotic-free EMEM (ATCC, 30–2003) supplemented with 10% FBS.

#### Human lung microvascular endothelial cells (MVEC)

Microvasular endothelial cells (ATCC, and Lonza) were expanded to passage 4 using either EGM^TM^-2 Microvascular Endothelial medium (Lonza) or Endothelial Cell Growth Medium (Promocell). Barrier function studies within PREDICT-96 devices used a 1:1 cell ratio of ATCC and Lonza-sourced MVEC.

### Barrier function *in vitro* assay

Barrier function was assessed using the PREDICT-96 dual channel microfluidic device as previously described [18, 19]. A schematic describing the system in detail is presented in S1 Fig. Human primary AT2 (derived using a protocol adapted from previous methods [20]) mono-cultures were established by seeding the device apical side twice at 70,000 cells/device (3 hours between seeds) in AT2 media: DMEM/F-12, with 2% FBS and 100 μg/mL Primocin. In co-cultures, MVEC were seeded on the device basal side at 20,000 cells/device in EC Medium: EGM^TM^-2 without VEGF, 100 μg/mL Primocin and 2% FBS. For 24-hour damage (Fig 2J and 2L), 20 ng/mL cytokines (IL-1β, TNFα, and IFNγ), 200 nM SARS-CoV-2 S protein RBD, or PBS were added in both AT2 and MVEC channels for 24-hours with MSC-EVs added concurrently and channels washed 24-hours after exposure. For chronic injury studies (Fig 2K and 2M), cells were exposed to 5 ng/mL cytokine mixture for 72-hours and MSC-EV added 24- or 48- hours following injury. Data are normalized to TEER immediately prior to cytokine damage (t = 0). Each condition was tested in 4–8 devices, across 2 PREDICT-96 plates. Included are representative data.

### SARS-CoV-2 RBD cell culture

The His-Tagged receptor binding domain (RBD) of the S1 SARS-CoV-2 spike protein (GenBank accession no. QHD43416.1; Arg319-Lys537; SPD-C52H3) was obtained from Acro Biosystems. Where indicated, Calu-3 cells were treated with 50 nM RBD and stimulated with the following tri-cytokine mix: 5 ng/mL TNFα (Proteintech, HZ-1014), 5 ng/mL IFNγ (Proteintech, HZ-1301), and 5 ng/ml IL1β (Proteintech HZ-1164).

### Flow cytometry

Calu-3 cells were stained with the following antibodies for flow cytometry 24 hours post MSC-EV treatment: ACE2 (AC18F; 30582 at 1:1000 dilution), from Cayman Chemicals, CD14 (M5E42; 555398 at 1:20 dilution) from BD, and 7-AAD (00-6993-50 at 1:10 dilution) from eBioscience. Cells were gated on live, single-cell populations. Calu-3 supernatant was collected 24 hours following MSC-EV treatment and analyzed for secreted cytokines and growth factors using LEGENDPlex (BioLegend, Human Cytokine Panel 2, 740102; HU Essential Immune Response Panel, 740929; HU Proinflammatory Chemokine Panel 1, 740984; Human Growth Factor Panel, 740180) according to the manufacturer’s protocols.

### Cell assays

Lactate dehydrogenase (LDH) secretion was analyzed 24 hours following MSC-EV treatment by calorimetry using CytoTox96 Non-Radioactive Cytotoxicity Assay (Promega, G1780) according to the manufacturer’s protocol. ACE2 activity was quantified 24 hours following MSC-EV treatment using ACE 2 Activity Assay Kit (PromoCell, PK-CA577-K897) according to the manufacturer’s protocols. Caspase-1, -3/7, and -8 activities were measured at 12-, 24-, or 48-hours following MSC-EV treatment using Promega CaspaseGlo kits and according to the manufacturer’s protocols (Caspase-1, G9953; Caspase-3/7, G8093; Caspase-8, G8202). HNE adducts were measured 24-hours after MSC-EV treatment by calorimetry using OxiSelect HNE Adduct Competitive ELISA Kit (Cell Biolabs, STA838). Cell death kinetics and endpoint permeability were determined using the RealTime-Glo Annexin V Apoptosis and Necrosis Assay (Promega, JA1012).

### Quantitative real-time PCR

Total mRNA was isolated from Calu-3 cell culture samples according to the manufacturer’s protocols using Qiagen RNeasy Mini Kit (74106). cDNA synthesis was completed using ABI High-Capacity cDNA Reverse Transcription Kit (4368813). qRT-PCR was performed with the QuantStudio^TM^ 6 System (Thermo Fisher) and TaqMan Fast Advanced Master Mix (4444965) according to the manufacturer’s protocols. qRT-PCR was completed using assay on demand primer-probe sets for ACE2 (Hs00222343_m1), MyD88 (Hs00182082_m1), TLR4 (Hs00152939_m1), and PPIA (Hs04194521_s1) as the housekeeping gene. Genes were standardized to PPIA and expressed as fold change relative to PBS-treated controls.

### Rat model of LPS-induced ALI

All *in vivo* experiments were approved by the Institutional Animal Care and Use Committee and were carried out by HDBiosciences. Sprague-Dawley rats (male, 150–200 g) were purchased from Shanghai BK Laboratory Animal Co., Ltd. The general condition (appearance and activity) of all animals was carefully monitored daily by the veterinarian. Animal body weights were recorded daily. Randomized adult rats (n = 10 for naïve, n = 12 for all other groups) were administered saline or 5 mg/kg intratracheal LPS (E. coli Serotype O55:B5, Sigma, Cat# L2880) on days 1 and 5 in-life. An intravascular bolus of PBS or MSC-EVs (200 μL) was given on day 1, three hours following LPS or on days 1, 3 and 5. All animals were allowed food and water ad libitum. On terminal day 6, whole-body plethysmography (FinePointe; Buxco/DSI) was utilized to measure breathing parameters and lung function in conscious, unrestrained rats. Rats were then anesthetized under pentobarbital and bronchoalveolar lavage fluid (BAL) was collected using a tracheal incision and 6 mL/rat saline. Blood was collected and serum processed from a cardiac puncture. Left lung was inflated with 10% NBF for histopathology evaluation. BAL and serum cytokines were evaluated using ELISA (Shanghai Jianglai, IL-6 Cat# JL20896, IL-1β Cat# JL20884, INF-γ Cat# JL13241, TNFα Cat# JL13202, TGF-β Cat# JL12342, CRP Cat# JL10640, RAGE Cat# JL21310, ANG-2 Cat# JL10293, IL-8 Cat# JL20898, IL-10 Cat# JL13427). Studies were performed and unbiased data were compiled by HDBiosciences. Lung injury score was evaluated at Visikol using algorithm-based automated image analysis. Two rats succumbed to unanticipated mortality after the second LPS challenge, one in the 50 pmol/kg and one in the 0.5 pmol/kg MSC-EV dose groups. All other animals maintained good health to the end of the study. All surgery was performed under pentobarbital anesthesia, and all efforts were made to minimize suffering.

### Statistical analysis

Values are expressed as mean ± SEM and analyzed using one-or two-way ANOVA with Tukey’s multiple comparisons test using GraphPad PRISM 8.3.

## Results

### Characterization of bone marrow MSC-derived extracellular vesicles

**Fig 1:**
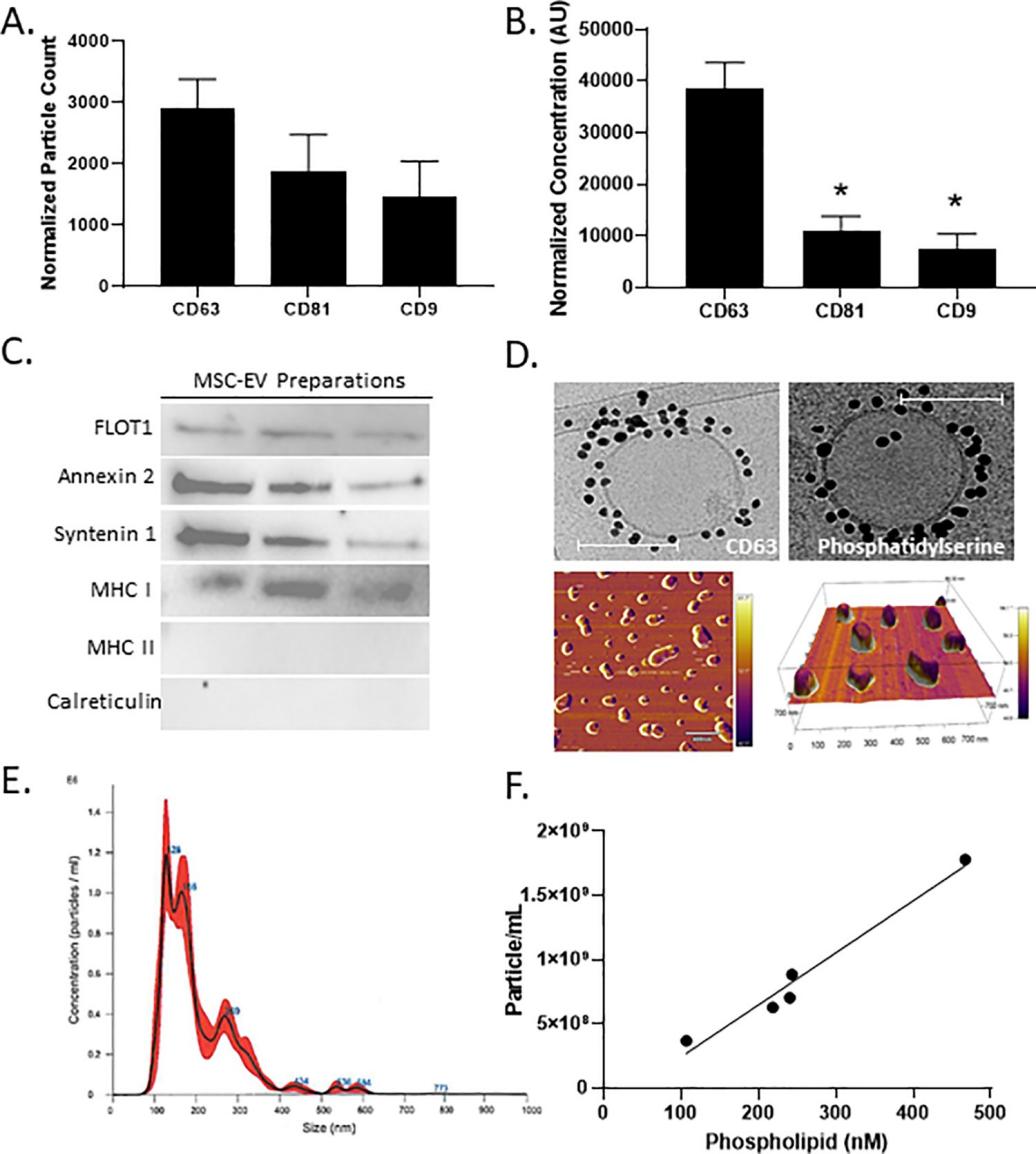
Isolated MSC-EVs demonstrate expression of known MSC-EV markers and contain phospholipid content correlating to particle dose. (A) Antibody microarray particle analysis of tetraspanin proteins. (B) Electrochemiluminescent multiplexed immunoassay detection of tetraspanin proteins. (C) Western blot analysis of MSC-EV proteins flotillin-1, Annexin-2, Syntenin-1, MHC-I and contaminating proteins MHC-II and calreticulin. (D) Immunogold cryo-transmission electron microscopy imaging for CD63 and phosphatidylserine. Atomic force microscopy for MSC-EV morphology and size. (E) Nanoparticle tracking for MSC-EV size and concentration. (F) MSC-EV particle density correlated to phospholipid content. Error bars are SEM of the mean. *p ≤0.05 vs CD63. Scale bar denotes 100 nm.

To characterize bone marrow MSC-derived extracellular vesicles (MSC-EVs), EV protein content, vesicle size, particle concentration, and phospholipid content were evaluated. Intact MSC-EV particles expressed tetraspanins CD63, CD81 and CD9, measured using affinity microarray technology functionalized with antibodies (Fig 1A). Similarly, a multiplex immunoassay was employed to elucidate protein abundance (Fig 1B), confirming tetraspanin presence and relative abundance. Isolated MSC-EVs contained EV-specific proteins flotillin-1, syntenin-1, annexin-2, and MHC-I class protein and were devoid of MHC-II and the apoptotic body marker calreticulin (Fig 1C). Likewise, the presence of MSC-EV surface markers CD63 and phosphatidylserine were confirmed using immunogold transmission electron microscopy. Further, immunogold imaging confirmed that MSC-EV vesicles are spherical or ovoid in shape and have a single lipid bilayer. These observations were reinforced using atomic force microscopy, analysis of which determined an average vesicle size of 150 nm (Fig 1D). Nanoparticle tracking confirmed that MSC-EVs range between 50 and 350 nm in size (Fig 1E), analysis of which revealed an average concentration of 1X10^9^ particles/mL. Lastly, the content of choline-containing phospholipids in isolated MSC-EVs was quantified using HPLC-MS (Fig 1F). Choline-containing phospholipids (e.g., phosphatidylcholine, sphingomyelin) are a structural foundation of MSC-EV membranes, making up 30–40% of lipid species [21]. MSC-EV particle concentration was found to correlate to MSC-EV phospholipid content (Fig 1F), and so this metric was used to determine MSC-EV dose in subsequent studies.

### Immunomodulatory impact of MSC-EV in cellular models of ALI

**Fig 2:**
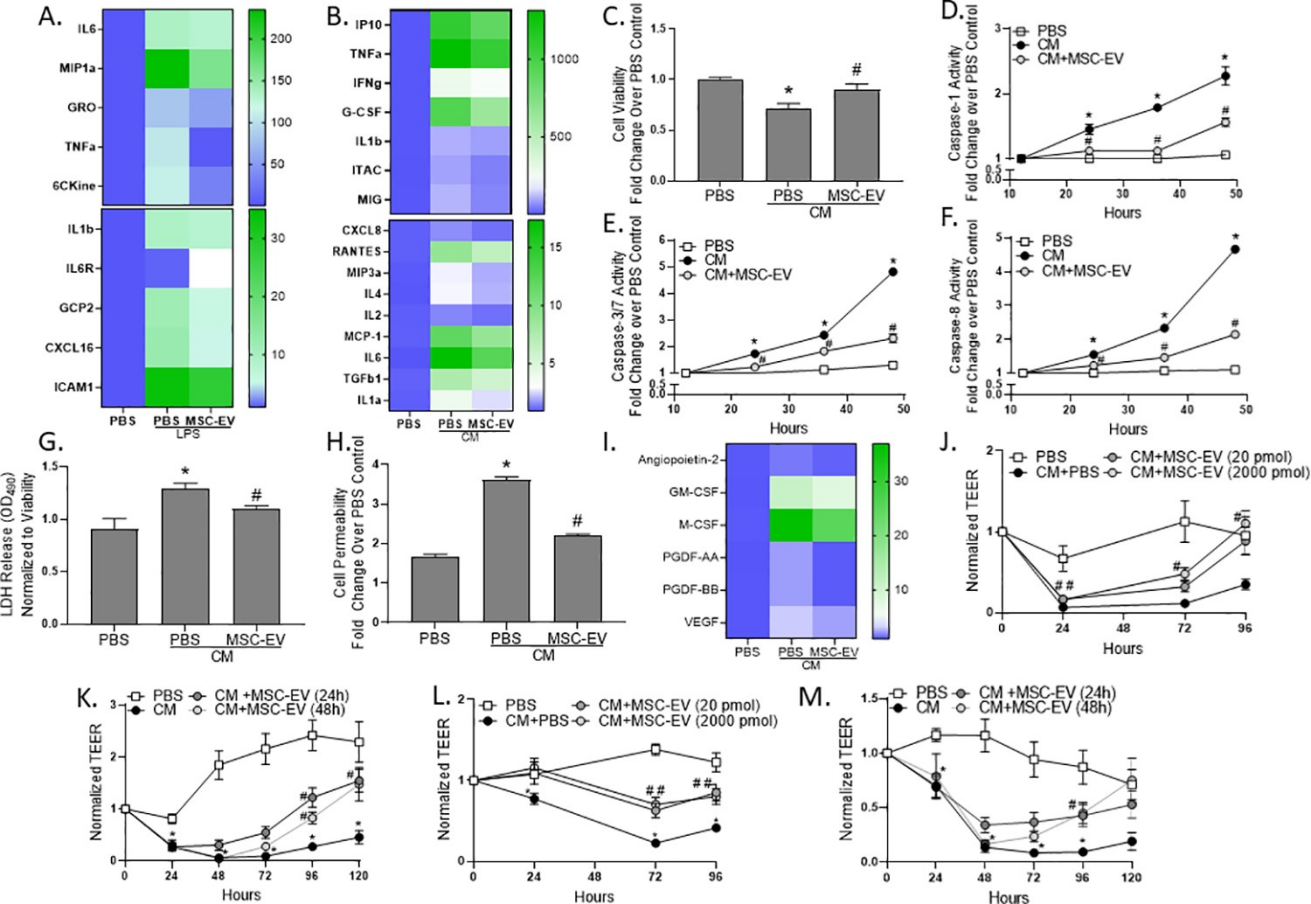
MSC-EVs decrease epithelial cell inflammation and improve cell health. Cytokine secretion was measured in (A) THP1 cell culture media after LPS administration and (B) calu-3 cell culture media after tri-cytokine addition (CM). Calu-3 cell viability was evaluated after a 24-hour CM exposure (C). Caspase-1 (D), caspase-3/7 (E), and caspase-8 (F) activities were evaluated in calu-3 cell lysates over time. LDH release into calu-3 culture media (G), cell permeability (H), and growth factor secretion into the media (I). Transepithelial/endothelial electrical resistance (TEER) was evaluated on primary human alveolar type 2 (AT2) after 24-hour (J) or 72-hour CM exposure (K). AT2 in co-culture with microvascular endothelial cells (MVEC) after 24-hour (L) or 72-hour CM exposure (M). Error bars are SEM of the mean. *p ≤0.05 vs PBS control; #p ≤0.05 vs CM.

An acute storm of pro-inflammatory cytokines, chemokines, and oxygen radicals, along with recruitment and activation of leukocytes, characterizes ALI clinical pathophysiology [22]. Depletion of circulating monocytes but not resident macrophages reduced the pulmonary inflammatory state in a mouse model of LPS-induced ALI [23]. To model monocyte activation, human THP-1 monocytes were pretreated with MSC-EVs prior to addition of LPS, a method commonly used to mimic ALI [24]. Exposure to LPS increased proinflammatory cytokine secretion, while pretreatment with MSC-EVs attenuated these levels (Fig 2A). However, while leukocytes represent a critical factor in ALI pathogenesis, it is the epithelium that acts as a first responder to injury and infection, regulating proinflammatory signals and thus leukocyte recruitment [25]. To simulate pro-inflammatory epithelium, Calu-3 human airway epithelial cells were evaluated after the addition of a tri-cytokine mix (CM) containing TNFα, IL1β and IFNγ. These cytokines were selected due to upregulation in the circulation of COVID-19 patients and established impact on the pro-inflammatory immune response [26]. As expected, the addition of CM to culture media increased pro-inflammatory cytokine secretion, while this response was mitigated by MSC-EVs (Fig 2B). The ALI pro-inflammatory environment contributes to alveolar epithelial cell damage, culminating in pyroptosis [27]. Cytokine exposure led to a loss of viable Calu-3 cells after 24 hours in culture, as measured by double stranded DNA exclusion, whereas MSC-EV prevented this cell loss (Fig 2C). To further explore this cellular salvage, apoptotic and pyroptotic signaling was evaluated over 48 hours of CM exposure. Caspase-1 activity, the established driver of pyroptosis, was elevated over 48 hours in culture with CM while MSC-EV tempered this activation over time (Fig 2D). Further, activity of caspases-3/7 (pro-apoptotic) and caspase-8 (pro-apoptotic, necrotic and pyroptotic) increased over time during CM exposure. Protein activity was partially reversed after MSC-EV treatment, indicative of an improvement in cellular health (Fig 2E, 2F). Supporting this hypothesis, lactate dehydrogenase (LDH) secretion, a cytosolic enzyme which is released into the extracellular space due to membrane damage, was elevated after 48 hours of CM exposure, mitigated by MSC-EV treatment (Fig 2G). Likewise, MSC-EV prevented the increase in cell permeability, a measure of membrane rupture and cellular death, after CM exposure (Fig 2H). Importantly, lung epithelial cells exposed to CM secreted PDGF and CSF, factors that correlate with ALI patient fibrosis [28] and mortality [29]. Further, angiopoitin-2, shown to trigger vascular regression, and VEGF, which promotes growth of immature, leaky vessels, were elevated in the culture media, and could contribute to endothelial cell damage and vascular leak [30]. MSC-EVs reduced these secreted factors, indicative of reduced cellular damage (Fig 2I).

Alveolar epithelial damage in ALI is further characterized by increased alveolar capillary permeability and edema, which ultimately lead to decreased lung compliance and hypoxemia [22]. To model alveolar capillary permeability, primary human alveolar type II (AT2) cellular barrier function was evaluated after the addition of CM using transepithelial/endothelial electrical resistance (TEER). Acute 24-hour CM addition, mimicking initial infection, decreased TEER compared to untreated control, resulting in a 2-fold difference at day 4 (96 hours) in culture. MSC-EV treatment, given concurrently with cytokine addition for the first 24 hours, partially rescued this loss in barrier function (Fig 2J). To mirror prolonged inflammation, AT2 cells were treated with CM for 72 hours, with MSC-EVs added 24 or 48 hours following the onset of inflammation. This cytokine regimen resulted in a dramatic decline in TEER which was salvaged in both MSC-EV treatment conditions (Fig 2K). To model the pulmonary-microvascular space, a co-culture system was established with epithelial AT2 cells on the apical side and human lung microvascular endothelial cells on the basal side of a microfluidic device. After acute 24-hour cytokine exposure, TEER was diminished in this co-culture system. MSC-EVs partially rescued the loss in barrier when treated concurrently with damage (Fig 2L). Persistent inflammation in the form of a 72-hour CM exposure resulted in a dramatic decline in barrier, reaching an 8-fold decrease at 48 hours compared to untreated control. MSC-EVs reversed the loss in barrier function when treated 24- or 48- hours after the onset of damage, normalizing TEER to control levels after 5 days when treated 48 hours after injury (Fig 2M). Further, MSC-EV treatment reduced cytokine secretion during acute and chronic CM co-culture (S2 Fig). No differences in cell viability or morphology were detected using calcien-AM viability stain after CM addition, indicating that the loss in TEER was due to barrier dysfunction and not cell loss (S3 Fig). These data are indicative of an MSC-EV-mediated improvement in barrier function during and after the onset of damaging inflammation. Because ALI contributes to morbidity and mortality associated with the ongoing SARS-CoV-2 pandemic, we sought to investigate the impact of MSC-EVs on a model of SARS-CoV-2-induced ALI.

### MSC-EVs normalize aberrant signaling in SARS-CoV-2 induced ALI

**Fig 3:**
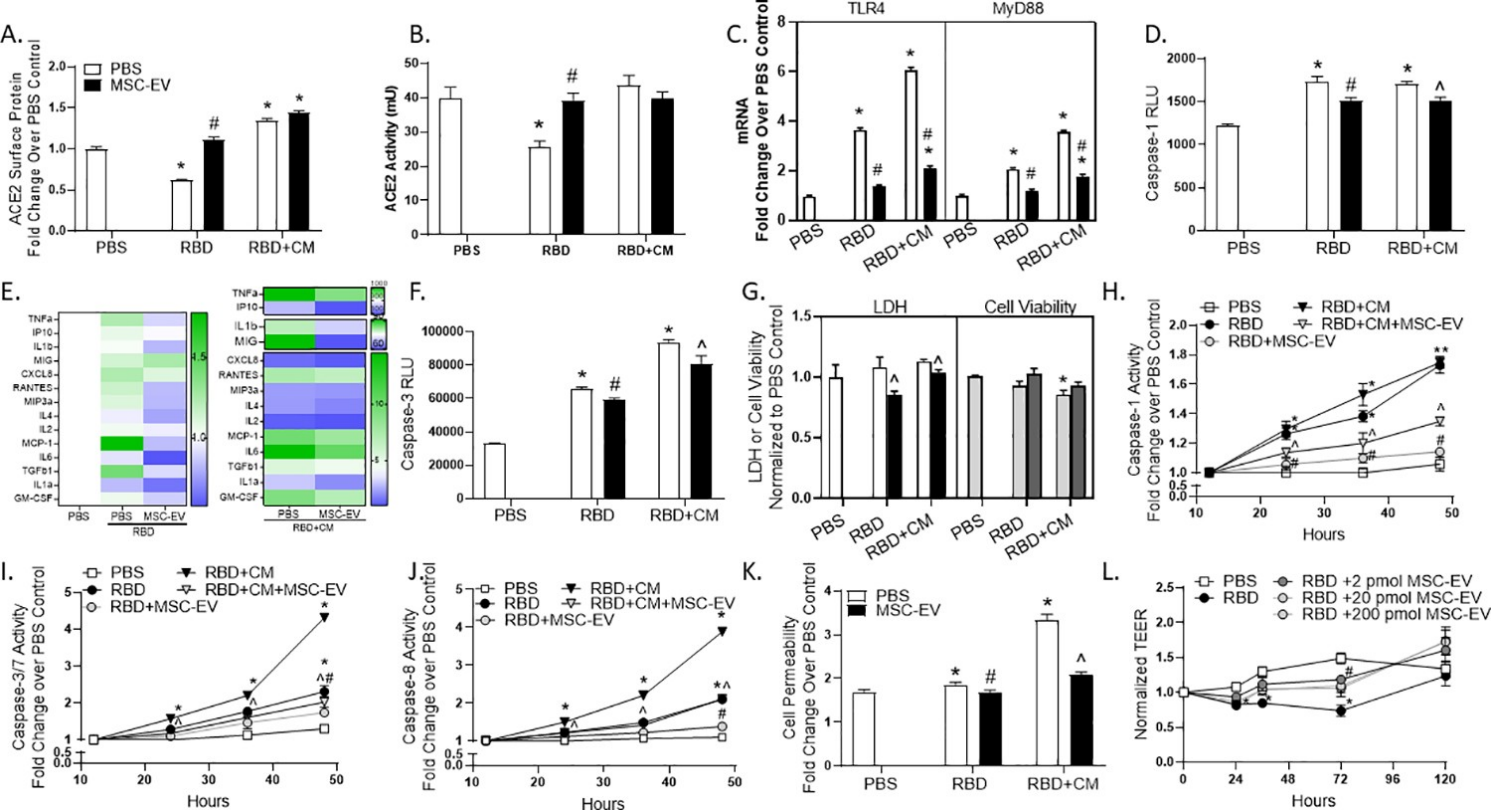
MSC-EVs improve ACE2 surface protein expression and cell health after SARS-CoV-2 exposure. Calu-3 cells were incubated with SARS-CoV-2 receptor binding domain (RBD) alone or in combination with tri-cytokine mix (CM). ACE2 surface protein (A) and activity (B), TLR4 and MyD88 gene expression (C), and caspase-1 activity (D) were evaluated in cells and cell lysates at 24 hours. Secreted cytokines (E) and cell lysate caspase-3 (F), LDH and cell viability (G) were evaluated at 24 hours. Activities of caspase-1, caspase-3/7, caspase-8 were evaluated in Calu-3 cell lysates over time (H), (I), (J). Cell permeability in Calu-3 lysates (K). Transepithelial/endothelial electrical resistance (TEER) was evaluated on primary human alveolar type 2 (AT2) and AT2 in co-culture with microvascular endothelial cells (MVEC; L). Error bars are SEM of the mean. *p ≤0.05 vs unstressed; #p ≤0.05 vs RBD; ^p ≤0.05 vs RBD+CM.

Due to the critical role of lung epithelium in COVID-19 disease progression [31], we established a lung airway epithelial cell model of SARS-CoV-2 ALI using Calu-3 cells and treatment with non-replicating SARS-CoV-2 receptor binding domain (RBD), added alone or in combination with a tri-cytokine mix containing TNFα, IL1β and IFNγ (RBD+CM). Treatment was evaluated with PBS control or MSC-EVs, added three hours following RBD or RBD+CM. As expected, RBD addition reduced ACE2 surface protein expression compared to PBS control (Fig 3A). The addition of the tri-cytokine mix together with RBD enhanced ACE2 expression compared to PBS control, supportive of ACE2 as an interferon-regulated gene [32]. Interestingly, MSC-EV treatment recovered ACE-2 surface protein compared to RBD alone. The impact of MSC-EV on ACE2 surface protein was not due to changes in mRNA (S4A Fig), indicating that MSC-EVs influence surface protein trafficking. To confirm ACE2 surface protein was reflective of active ACE2 protein, ACE2 activity was evaluated. Reinforcing Fig 3A, while RBD reduced ACE2 activity compared to PBS control, MSC-EV normalized these levels (Fig 3B). To determine the effect of MSC-EVs on additional viral response proteins, CD14 was investigated. RBD and RBD+CM increased CD14 surface protein compared to PBS control, while MSC-EVs reduced CD14 protein to a level comparable to PBS control (S4B Fig). Angiotensin II, the substrate of ACE2, is a known inducer of TLR4 which signals through MyD88 to promote inflammation and cell death [33–35]. In support of these data, qRT-PCR measurements revealed an increase in TLR4 mRNA with RBD treatment over PBS control. MSC-EVs blunted this response, reducing TLR4 mRNA by 60% and 65% in RBD and RBD+CM conditions, partially normalizing mRNA levels to that of PBS control. Similarly, RBD increased MyD88 mRNA, a response which was intensified by the addition of CM. Consistent with the reduction in TLR4 signaling, MSC-EVs reduced MyD88 mRNA compared to control (Fig 3C).

We next sought to determine if MSC-EV treatment would modulate caspase-related inflammasome activation downstream of the TLR4/MyD88 axis. RBD increased expression of the inflammasome-associated caspase-1, reinforcing literature evidence that SARS-CoV-2 activates caspase-1 [33, 38]. MSC-EVs blunt this activation, reducing caspase-1 activity in both treatment conditions (Fig 3D). Consistent with inflammatory activation, an increase in cytokine secretion was observed after exposure to RBD, a response that was exacerbated by the addition of CM. MSC-EVs reduced secretion of these cytokines, indicative of anti-inflammatory activity (Fig 3E). Cytokine production and oxidative stress combine to exacerbate the pathogenesis of SARS-CoV-2 [36]. In support of these data, 4-hydroxynonenal (4-HNE), a measure of lipid peroxidation, increased with the addition of RBD and trended to increase with RBD+CM. MSC-EVs reduced these levels, normalizing each group to that of the PBS control (S4C Fig). Further, while RBD increased caspase-3 activity, a phenotype which was exacerbated by CM addition, MSC-EVs partially normalize function (Fig 3F). Similarly, RBD and RBD+CM trended to increase LDH secretion and decrease cell viability compared to control. This response was slight, likely because activated apoptotic signaling at this acute timepoint did not yet result in dramatic loss of viable cells. Nonetheless, MSC-EVs decreased secreted LDH and trended to improve cell viability (Fig 3G). To thoroughly investigate the impact of SARS-CoV-2 on cell health, Calu-3 cells were evaluated every 12 hours during a 48-hour exposure to RBD or RBD+CM. Extended RBD and RBD+CM exposure increase caspase-1 activity over time, a response which was tempered after MSC-EV addition (Fig 3H). RBD increased activities of caspases-3/7 and -8 over time, made more robust with the addition of CM. MSC-EV treatment decreased these pro-apoptotic protein activities (Fig 3I and 3J). Fortifying these data, MSC-EV blocked the increase in cell permeability after RBD or RBD+CM, indicative of cellular salvage (Fig 3K). Supporting the impact of MSC-EV on cellular salvage, MSC-EV were similarly effective at reducing cell loss and caspase signaling after CM alone (S4D-S4I Fig). Lastly, a co-culture system with epithelial AT2 cells on the apical side and human lung microvascular endothelial cells on the basal side was used to evaluate cell barrier function in the context of RBD. While RBD addition reduced cell barrier function compared to control, MSC-EV partially restored these levels (Fig 3L). In aggregate, these data are suggestive of a protective effect of MSC-EVs on lung epithelium after SARS-CoV-2 infection.

### MSC-EV treatment improves lung injury in a rat model of ALI

**Fig 4:**
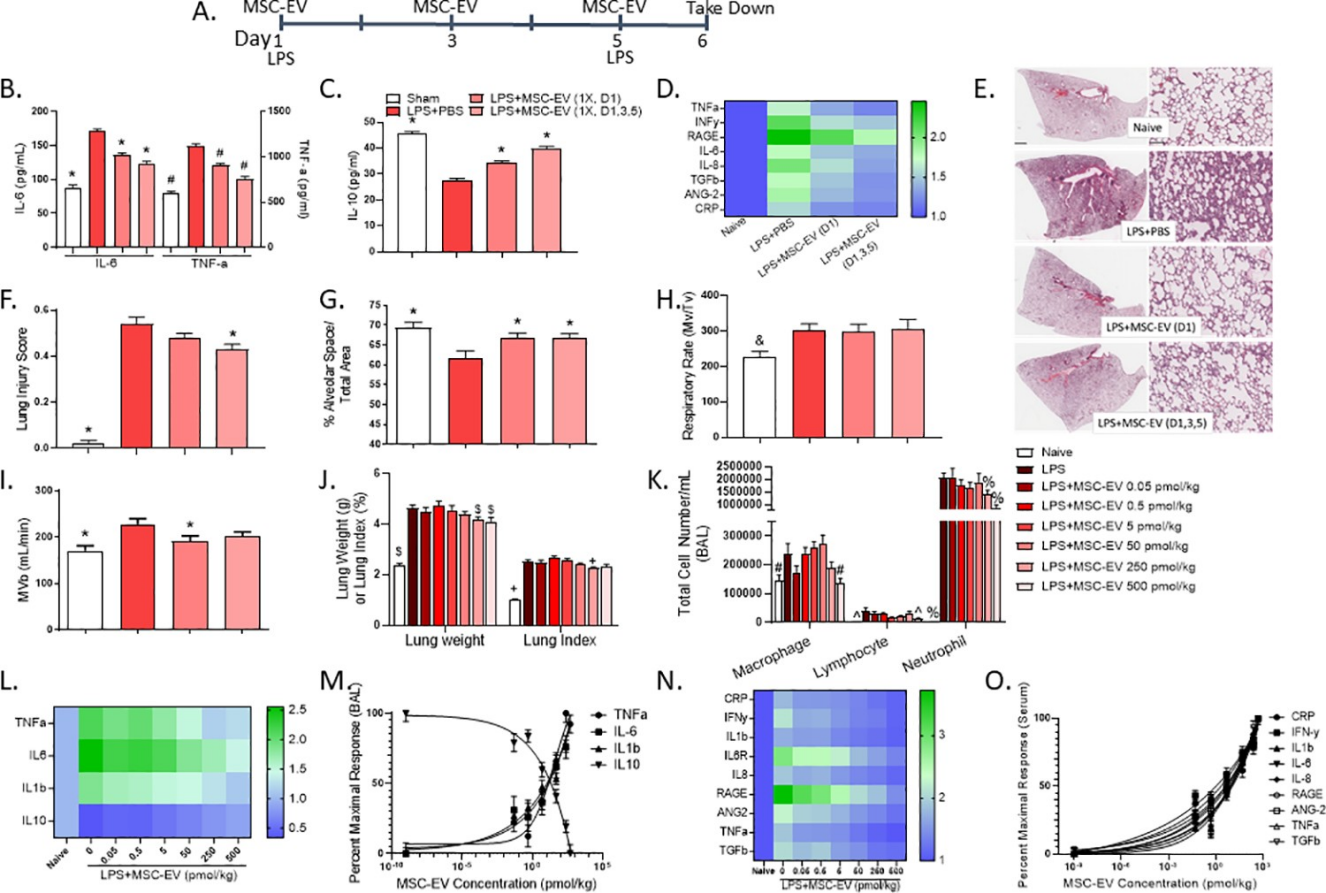
MSC-EV therapy improves ALI in a rat model of the disease. (A) Schematic of the LPS-induced rat model of ALI depicting LPS intratracheal installation and MSC-EV intravascular administration. (B) IL-6, TNFα and (C) IL-10 were measured in bronchoalveolar lavage fluid (BAL). (D) Serum cytokine levels normalized to naïve control. (E) histology lung scans (left panel) with representative magnification (right panel). Histology scans were used to calculate (F) Lung injury score and (G) percent alveolar space. Scale bar denotes 2 mm (left panel) and 100 μm (right panel). Plethysmography (H) respiratory rate and (I) minute volume. (J) lung weight and lung index. (K) infiltrating cells from the BAL. (L) BAL cytokines normalized to naïve control. (M) MSC-EV percent maximal response in the BAL. (N) serum cytokines normalized to naïve control. (O) MSC-EV percent maximal response in serum. Error bars are SEM of the mean. * p<0.05 vs LPS (cytokine, lung function); & p<0.1 vs LPS (lung function) $ p<0.05 vs LPS (lung weight); + vs LPS (lung index) # P<0.05 vs LPS (Macrophage); ^ P<0.05 vs LPS (Lymphocyte); % P<0.05 vs LPS (Neutrophil).

To investigate therapeutic potential, we evaluated the impact of MSC-EV treatment in a rat model of LPS-induced ALI. ALI was induced via intratracheal administration of LPS on days 1 and 5. Intravenous MSC-EVs were given 3 hours following LPS administration on day 1 or on days 1, 3 and 5 (Fig 4A). LPS administration increased IL-6 and TNFα pro-inflammatory cytokines in the bronchoalveolar lavage fluid (BAL). MSC-EVs partially reversed accumulation of these cytokines, with multiple doses appearing more effective than a single administration (Fig 4B). Further demonstrating the immunomodulatory function of MSC-EVs, levels of anti-inflammatory IL-10 were diminished in the BAL after LPS administration, partially reversed after MSC-EV treatment (Fig 4C). Moreover, MSC-EVs blunted LPS-induced cytokine secretion in the serum at each dose frequency tested (Fig 4D). These data mirror in vitro cytokine analysis (Figs 2 and 3), demonstrating an immunomodulatory impact of MSC-EV, both in vitro and in vivo.

Paralleling clinical ALI, exposure to LPS caused alveolar damage characterized by excessive inflammation in the alveolar interstitium and airspaces ([37, 38]; Fig 4E). MSC-EV treatment mitigated this injury, as shown via improved histological appearance, reduced lung injury score, and increased alveolar space (Fig 4E–4G). Respiratory rate and minute volume were increased following LPS exposure, reflective of increased respiratory drive in ALI patients [38, 39]. While MSC-EV administration did not impact respiratory rate, a decrease in minute volume was observed compared to LPS control with the single dose administration reaching statistical significance (Fig 4H and 4I). These data may suggest an MSC-EV- mediated improvement in lung function following onset of ALI.

Because a single dose achieved similar immunomodulatory, histological, and functional improvements as the multi-dose paradigm, subsequent studies sought to explore the pharmacodynamic effects of MSC-EVs using one administration and a wide range of doses. LPS exposure increased lung weight and index compared to naïve control, consistent with disruption of the alveolar-capillary barrier. MSC-EVs improved this phenotype at 250 and 500 pmol/kg for lung weight and 250 pmol/kg for lung index (Fig 4J). Further, LPS administration increased infiltrating macrophages, lymphocytes and neutrophils in the BAL. Consistent with BAL taken from ALI patients, neutrophil infiltration accounted for the largest differential [39]. MSC-EVs again improved these outcomes at the highest doses tested (Fig 4K). These data are indicative of an MSC-EV dose-responsive improvement in lung injury. Further, while LPS increased pro-inflammatory and decreased anti-inflammatory cytokines in the BAL, MSC-EV treatment reversed this phenotype (Fig 4L). The dose kinetics, graphed as percent improvement over LPS control, highlight a dose-responsive pattern (Fig 4M). Similarly, LPS triggered pro-inflammatory cytokine secretion into the circulation, and MSC-EV blunted this effect (Fig 4N). Once more, the treatment effect of MSC-EVs was robust and dose-responsive (Fig 4O). Taken together, these data suggest clinical relevance for MSC-EVs in ALI.

## Discussion

The current study sought to explore the treatment benefit of MSC-EVs in COVID-19-induced ALI. We provide evidence of an immunomodulatory benefit and protection from alveolar-capillary barrier damage in epithelial cells. Further, we demonstrate that MSC-EVs alter SARS-CoV-2 signaling in vitro, suggestive of a treatment benefit in patients with ALI. To test this hypothesis in vivo, we examined the impact of MSC-EV in a rat model of ALI and established a robust, dose-responsive improvement in lung inflammation and edema. In aggregate, our results suggest that MSC-EVs may benefit patients with ALI, inclusive of ALI caused by COVID-19.

An ongoing global threat, COVID-19 is caused by viral SARS-CoV-2. The SARS-CoV-2 receptor binding domain (RBD) binds host cell angiotensin-converting enzyme 2 (ACE2), which is highly expressed in the lung epithelium [40, 41]. Activation of lung epithelium is paramount to the viral host response, regulating the innate and adaptive immune system [24]. Because human lung Calu-3 cells express ACE2 protein and are susceptible to SARS-CoV-2 infection [42], we selected this cell line to capture the SARS-CoV-2 phenotype *in vitro*. Further, we utilized treatment with the replication incompetent SARS-CoV-2 receptor binding domain (RBD) to model viral infection. Consistent with COVID-19 pathogenesis, exposure to RBD decreased ACE2 surface expression in Calu-3 cells compared to control ([43], Fig 3A). Further, data herein mirror recent reports demonstrating that SARS-CoV-2 virus activates pyroptotic and apoptotic signaling in airway epithelium to stimulate a downstream immune response ([31, 34, 44, 45], Fig 3). While RBD treatment and the subsequent decrease in ACE2 surface protein does not fully capture the complexity of COVID-19-induced ALI, these data support the use of RBD and Calu-3 cells as a model of infection. Importantly, treatment with MSC-EVs prevented the decline in ACE2 surface protein, providing evidence that MSC-EVs can benefit host protein signaling integral to the SARS-CoV-2 infection.

An unexplored explanation for the MSC-EV-mediated increase in ACE2 surface protein after exposure to RBD is a direct competition of MSC-EVs with SARS-CoV-2 for ACE2 binding and uptake. Recent reports suggest that reducing SARS-CoV-2 binding on ACE2 could account for global improvements in COVID-19 pathology [46]. More work is needed to investigate this potential mechanism. Notwithstanding, it has been hypothesized that MSC-EVs engineered to express ACE2 could couple the reparative efficacy of MSC-EVs with a SARS-CoV-2 binding sink [47]. In this paradigm, ACE2 expression may enhance MSC-EVs and act as a next generation therapy in the treatment of COVID-19.

The lack of a rodent ACE2 human equivalent limits the ability to precisely recapitulate SARS-CoV-2 viral infection in a non-clinical model [14]. Because of this limitation, a rat model of LPS-induced ALI was used to characterize the pharmacodynamic effect of MSC-EVs on ALI progression and symptom management. LPS was given in two injections over a 6-day in-life period. This LPS regimen was selected due to its impact on lung architecture, consistent with human ALI [48]. Further, the LPS model herein features necessary characteristics of ALI agreed upon by the American Thoracic Society [49]. Namely, LPS exposure increased histological evidence of tissue injury (thickening of alveolar wall), inflammatory response (BAL neutrophil infiltration and proinflammatory cytokines, Fig 4), and physiological dysfunction (increased respiratory rate and minute ventilation, Fig 4). Thus, improvement in these features via MSC-EV treatment carries weight in predicting its clinical utility.

The concept of MSC-EVs for the treatment of COVID-19-induced ALI is not new. MSC-EVs have demonstrated efficacy in several preclinical models of ALI [9]. Further, ongoing clinical trials will examine the safety and impact of MSC-EVs in patients with COVID-19 [13]. However, direct evidence of MSC-EV efficacy is lacking. Current treatments of COVID-19 may shed light on the utility of EVs in the clinic. Indeed, convalescent blood products have been suggested to act in part through delivery of plasma-associated extracellular vesicles [13]. In support of this hypothesis, data herein demonstrate a role for MSC-EVs in reducing cellular inflammation and protecting the alveolar-epithelial barrier in non-clinical ALI.

Taken together, we provide support for the use of MSV-EVs for the treatment of ALI, including ALI resulting from COVID-19 infection. Clinical MSC-EVs at doses of 250 pmol/kg phospholipid or greater are predicted to reduce local and systemic inflammation and lung injury.

## Supporting information

S1 Fig Organ-on-chip vascularized alveolar model schematic. The platform comprises of 96 independent tissue models each containing two independent microfluidic channels separated by a permeable membrane (A), on a tissue culture microplate in a standard microplate format, the micro-pump sensing array lid consisting of 192 micropumps and 384 electrodes integrated into the plate lid, and the user-programmable system controller unit (B). The configuration of the channels allows for flexible co-culture of various cell types on either side of the membrane to develop complex tissue models. In this alveolar model, AT2 (primary human alveolar type 2 pneumocytes) tissue is generated in the top channel and lung microvascular (human primary lung microvascular endothelial cells, MVEC) tissue in the bottom channel. MVEC tissue is generated in the bottom channel by flow conditioning for 4 days at low (~0.5 dynes/cm^2^) shear. Bottom IF image shows healthy flow path-aligned MVEC barrier tissue (4 d culture). Alveolar tissue (AT2-dominant) is then generated in the top channel under static (no flow) conditions. IF images show AT2 character (HT-280) and junctional formation (ZO-1) after 4 days culture (C). This alveolar-microvascular model maintains stable, high TEER barrier for roughly a week after TEER plateau (D).
(TIF)

S2 Fig MSC-EVs decrease inflammation in PREDICT96 microphysiological alveolar-microvascular model. Cytokine secretion was measured in PREDICT96 alveolar-microvascular co-cultures at various timepoints (24h, 48h, or 96h) after CM addition only (-) or CM and MSC-EV addition (+). Damage regimes evaluated were 24 hr cytokine injury and addition of MSC-EVs at same time of cytokine injury (A, acute response), or delayed MSC-EV addition after 24 hr cytokine injury (B, chronic response) and continued cytokine addition for 72 hr total. Scales are normalized to cytokine control at each timepoint.
(TIF)

S3 Fig Cytokine treatment does not impact cellular coverage or morphology in the PREDICT96 alveolar-microvascular model. Representative device images of live alveolar cells (calcein-AM stained, green) at 96 hr post-treatment (or untreated control) in 72h-treated/24h washout tri-cytokine devices (A), 24h-treated/72h washout cytokine devices B), and untreated devices (C). Specific TEER for each device displayed in white. Total TEER of co-cultures is dominated by AT2 contribution. Scale bar equals 200 μm.
(TIF)

S4 Fig MSC-EVs improve cell health after exposure to cytokine mix. Calu-3 cells were incubated with SARS-CoV-2 receptor binding domain (RBD) alone or in combination with tri-cytokine mix (CM). ACE2 mRNA (A) CD14 surface protein (B) and 4-HNE protein adducts (C) were evaluated. LDH (D) and cell permeability (E) were evaluated in calu-3 cells incubated with tri-cytokine mix (CM) alone. Activities of caspase-1, caspase-3/7, caspase-8 were evaluated in CM-treated calu-3 lysates over time (F-H). Error bars are SEM of the mean. *p ≤0.05 vs unstressed; #p ≤0.05 vs CM.
(TIF)

S1 Raw images (PDF)
