# Supplementary figures and images for: Mesenchymal stromal cell-derived extracellular vesicles reduce lung inflammation and damage in nonclinical acute lung injury: Implications for COVID-19
Source: PLoS One. 2021 Nov 15;16(11):e0259732. doi: 10.1371/journal.pone.0259732 (PMC8592477; doi:10.1371/journal.pone.0259732)

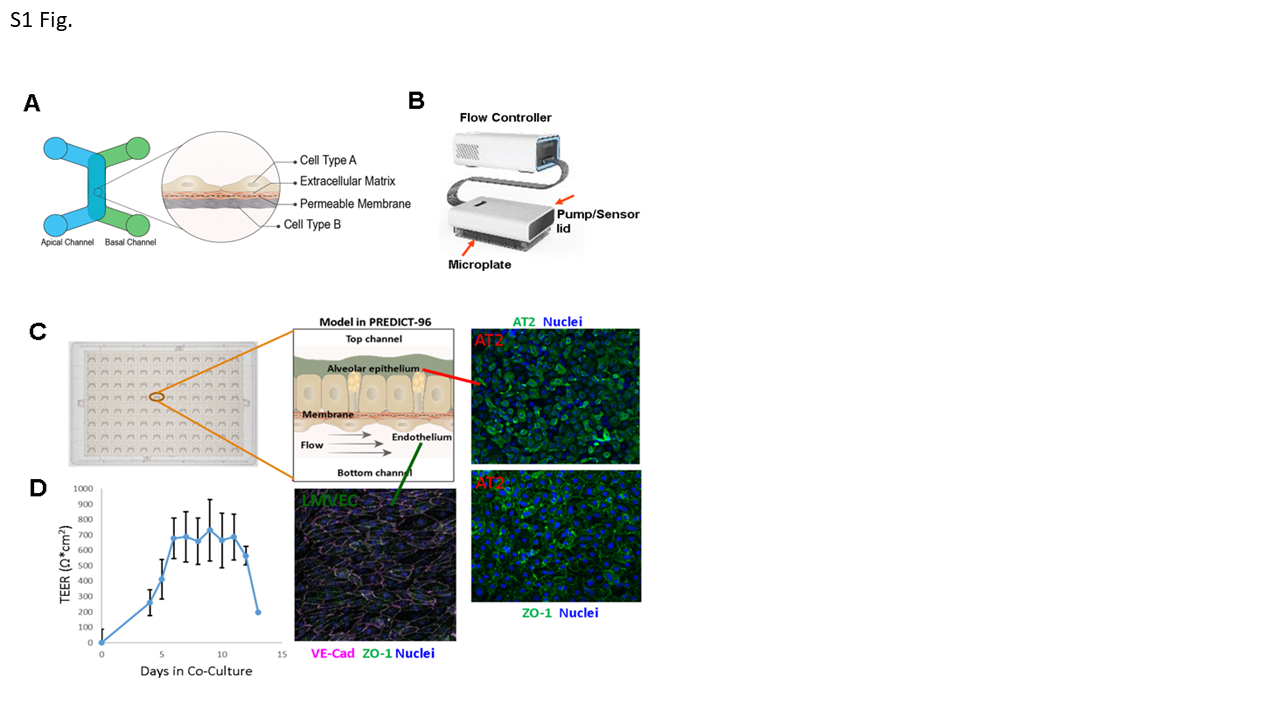

Supplement: S1 Fig — The platform comprises of 96 independent tissue models each containing two independent microfluidic channels separated by a permeable membrane (A), on a tissue culture microplate in a standard microplate format, the micro-pump sensing array lid consisting of 192 micropumps and 384 electrodes integrated into the plate lid, and the user-programmable system controller unit (B). The configuration of the channels allows for flexible co-culture of various cell types on either side of the membrane to develop complex tissue models. In this alveolar model, AT2 (primary human alveolar type 2 pneumocytes) tissue is generated in the top channel and lung microvascular (human primary lung microvascular endothelial cells, MVEC) tissue in the bottom channel. MVEC tissue is generated in the bottom channel by flow conditioning for 4 days at low (~0.5 dynes/cm2) shear. Bottom IF image shows healthy flow path-aligned MVEC barrier tissue (4 d culture). Alveolar tissue (AT2-dominant) is then generated in the top channel under static (no flow) conditions. IF images show AT2 character (HT-280) and junctional formation (ZO-1) after 4 days culture (C). This alveolar-microvascular model maintains stable, high TEER barrier for roughly a week after TEER plateau (D). (TIF) [file pone.0259732.s001.tif]

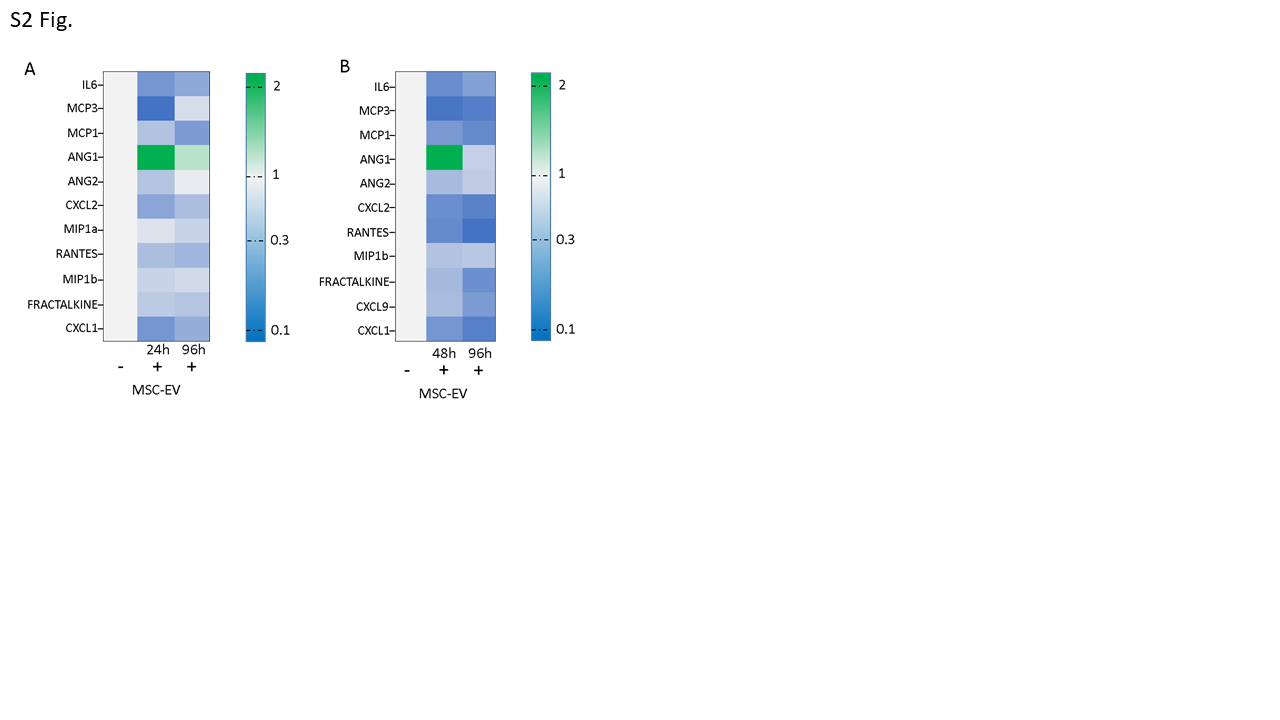

Supplement: S2 Fig — Cytokine secretion was measured in PREDICT96 alveolar-microvascular co-cultures at various timepoints (24h, 48h, or 96h) after CM addition only (-) or CM and MSC-EV addition (+). Damage regimes evaluated were 24 hr cytokine injury and addition of MSC-EVs at same time of cytokine injury (A, acute response), or delayed MSC-EV addition after 24 hr cytokine injury (B, chronic response) and continued cytokine addition for 72 hr total. Scales are normalized to cytokine control at each timepoint. (TIF) [file pone.0259732.s002.tif]

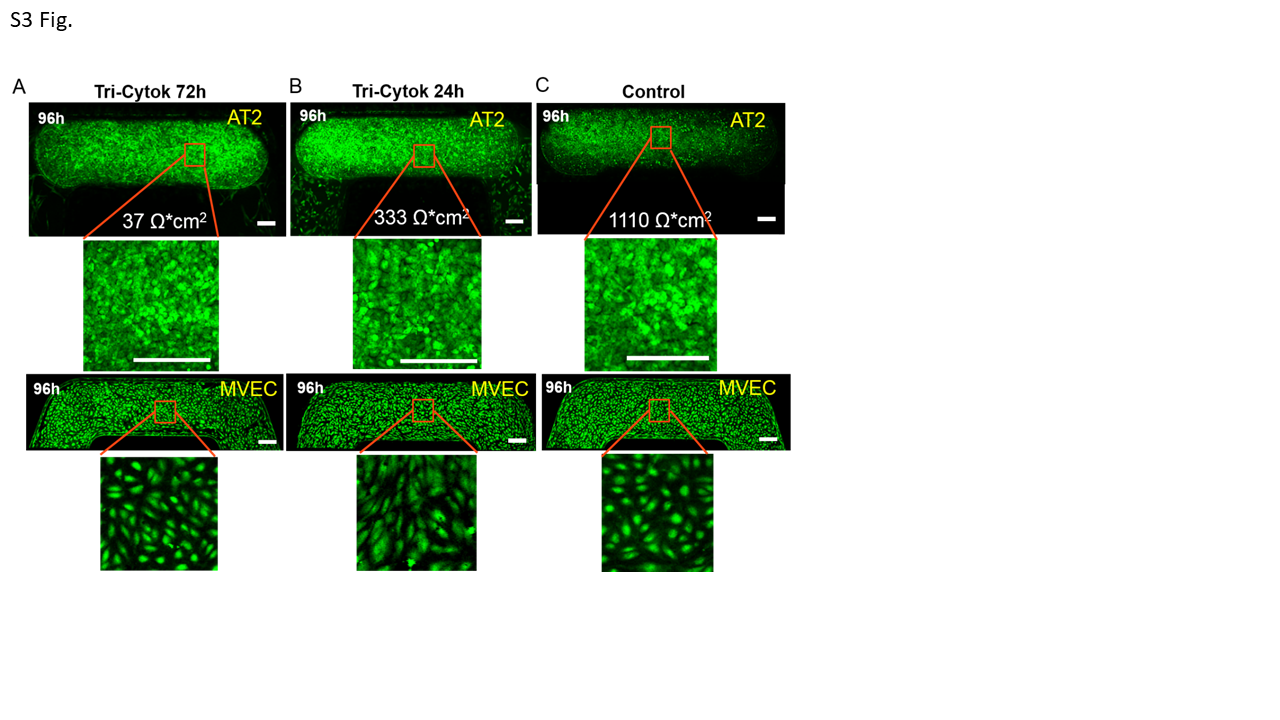

Supplement: S3 Fig — Representative device images of live alveolar cells (calcein-AM stained, green) at 96 hr post-treatment (or untreated control) in 72h-treated/24h washout tri-cytokine devices (A), 24h-treated/72h washout cytokine devices B), and untreated devices (C). Specific TEER for each device displayed in white. Total TEER of co-cultures is dominated by AT2 contribution. Scale bar equals 200 μm. (TIF) [file pone.0259732.s003.tif]

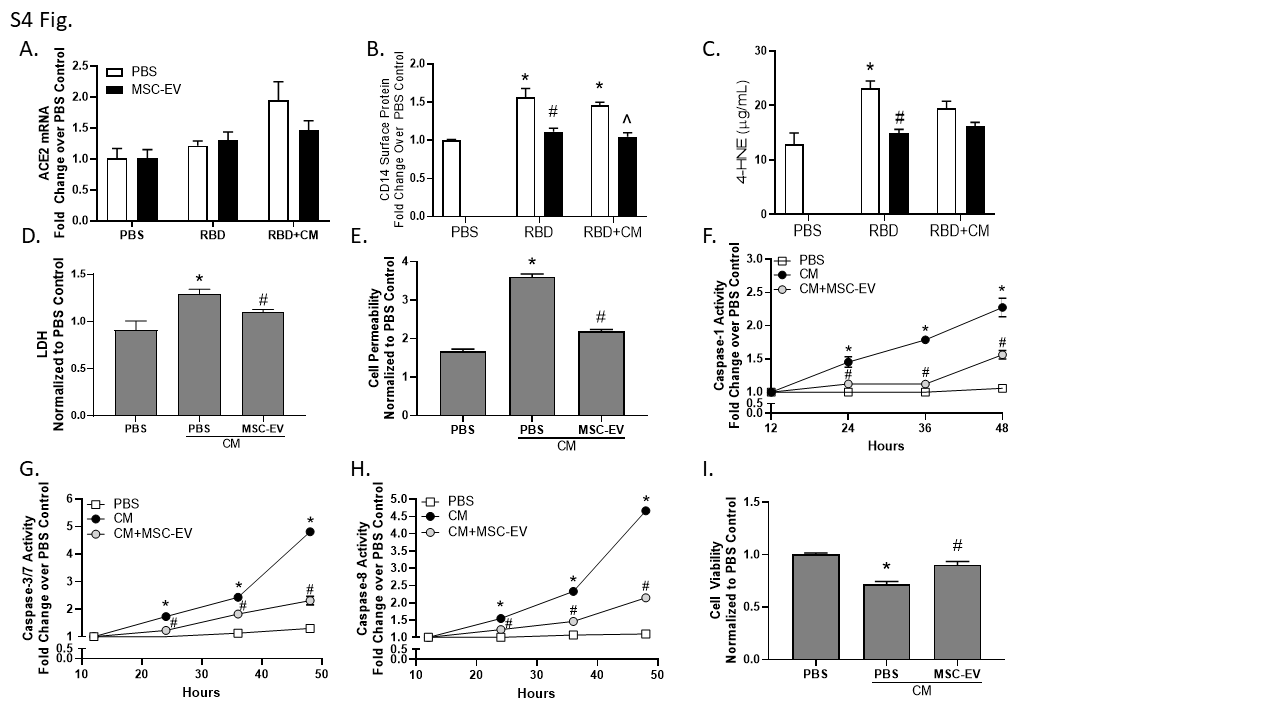

Supplement: S4 Fig — Calu-3 cells were incubated with SARS-CoV-2 receptor binding domain (RBD) alone or in combination with tri-cytokine mix (CM). ACE2 mRNA (A) CD14 surface protein (B) and 4-HNE protein adducts (C) were evaluated. LDH (D) and cell permeability (E) were evaluated in calu-3 cells incubated with tri-cytokine mix (CM) alone. Activities of caspase-1, caspase-3/7, caspase-8 were evaluated in CM-treated calu-3 lysates over time (F-H). Error bars are SEM of the mean. *p ≤0.05 vs unstressed; #p ≤0.05 vs CM. (TIF) [file pone.0259732.s004.tif]
