# Supplementary material for: Mesenchymal stromal cell-derived extracellular vesicles reduce lung inflammation and damage in nonclinical acute lung injury: Implications for COVID-19
Source: PLoS One. 2021 Nov 15;16(11):e0259732. doi: 10.1371/journal.pone.0259732 (PMC8592477; doi:10.1371/journal.pone.0259732)
Supplement: S1 Raw images — (PDF) [file pone.0259732.s005.pdf]

FLOT1 Full Blot:expected 47 kDa

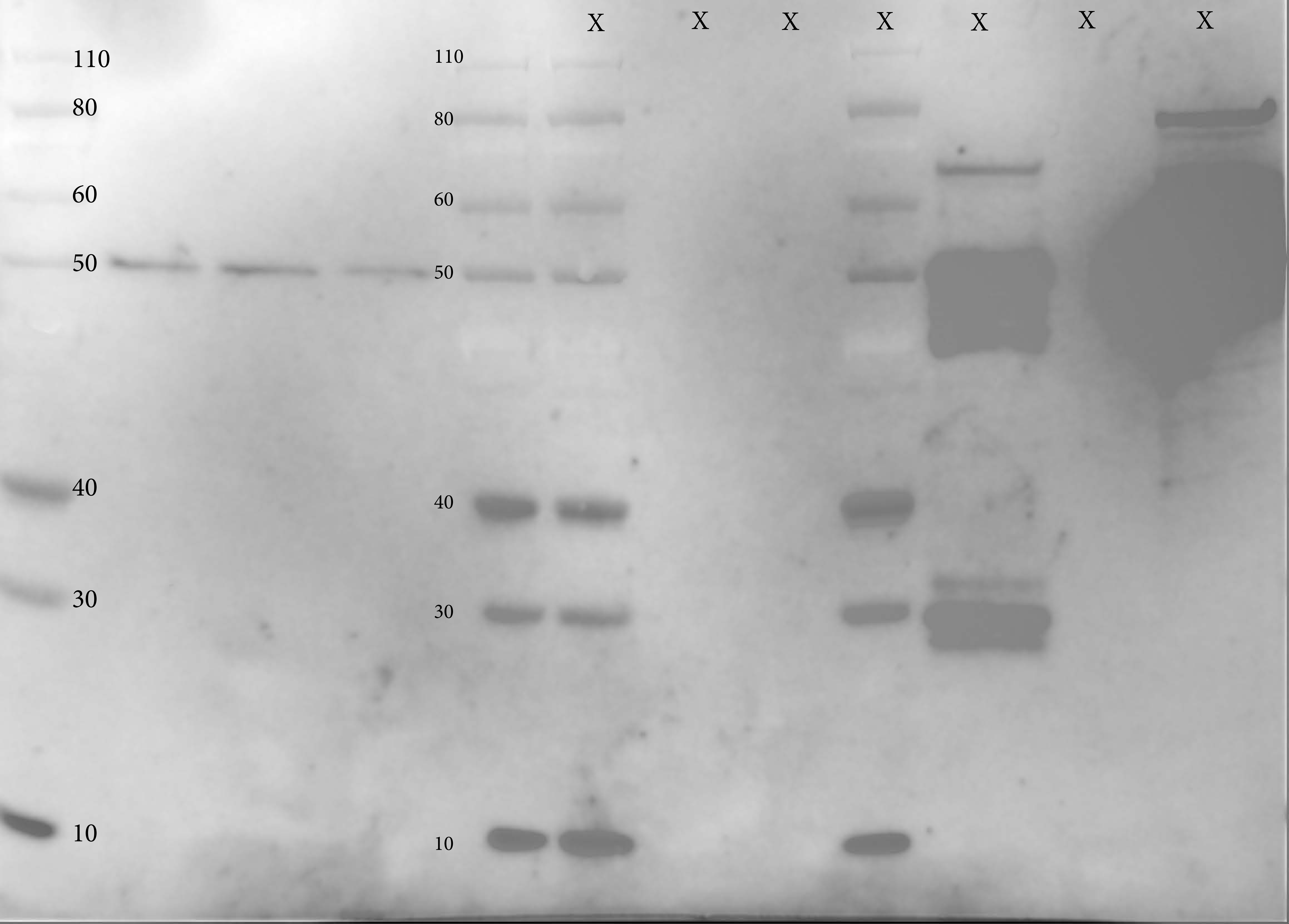

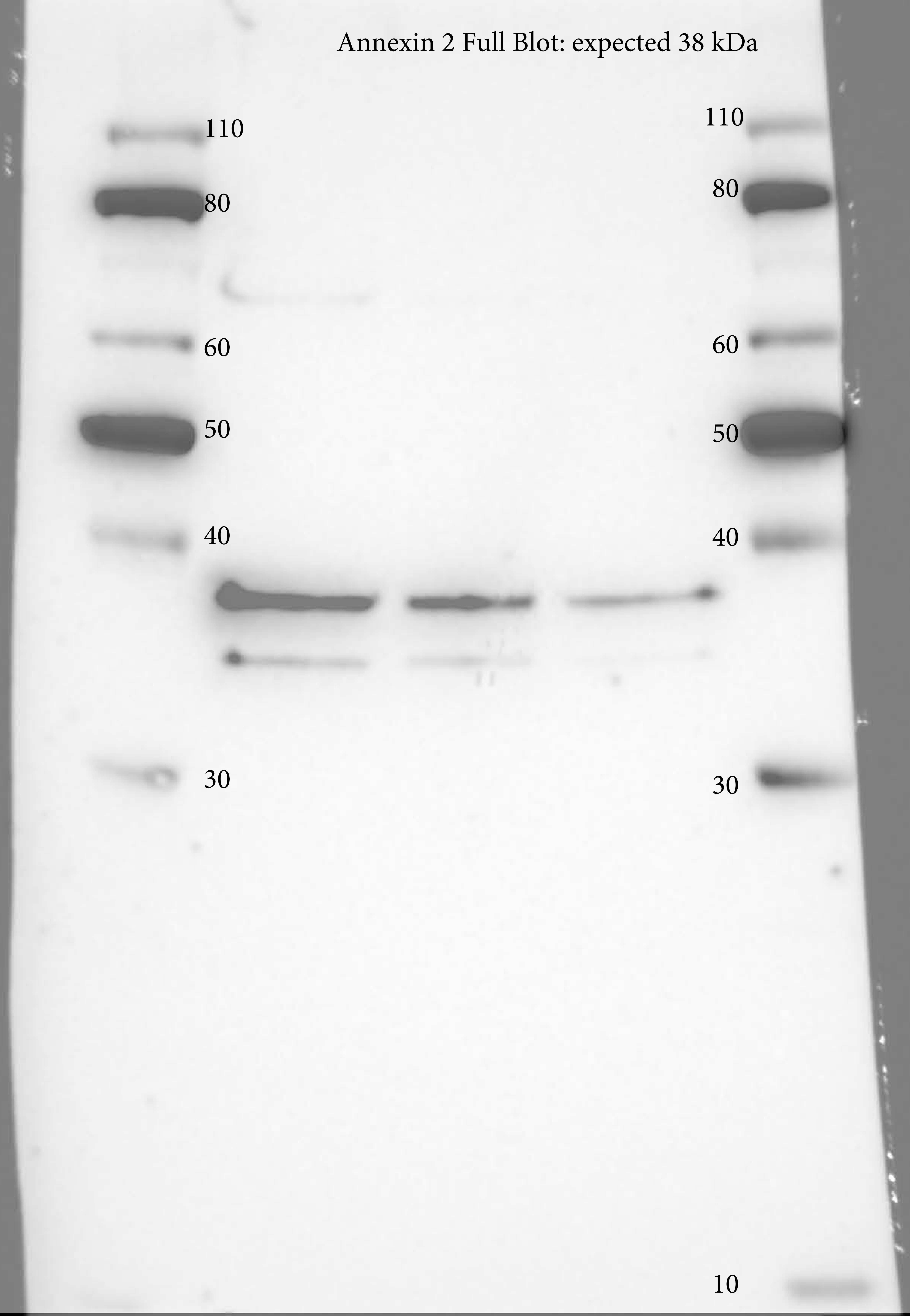

Annexin 2 Full Blot: expected 38 kDa
110
80
60
50
40
30
110
80
60
50
40
30
10

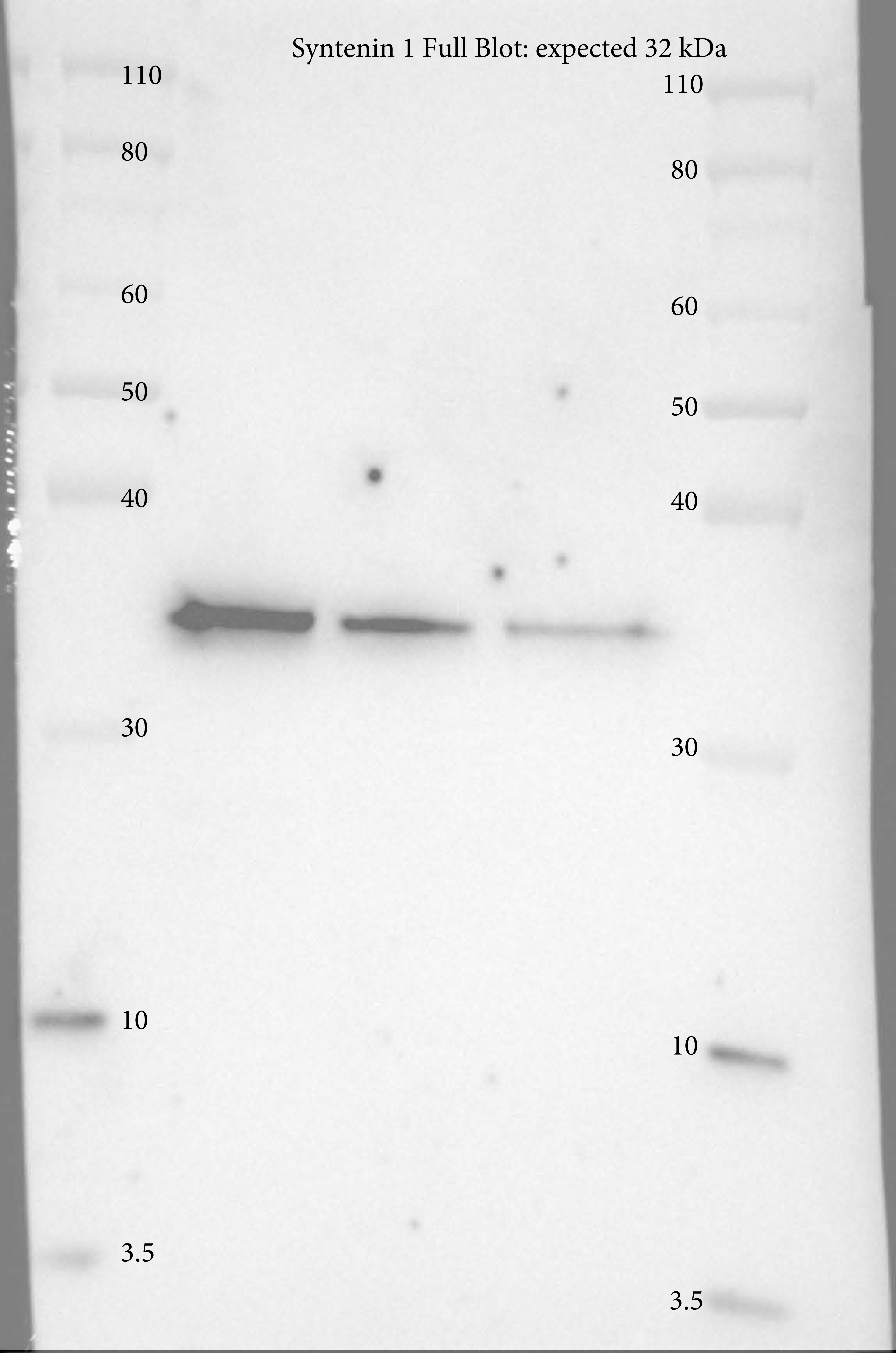

Syntenin 1 Full Blot: expected 32 kDa

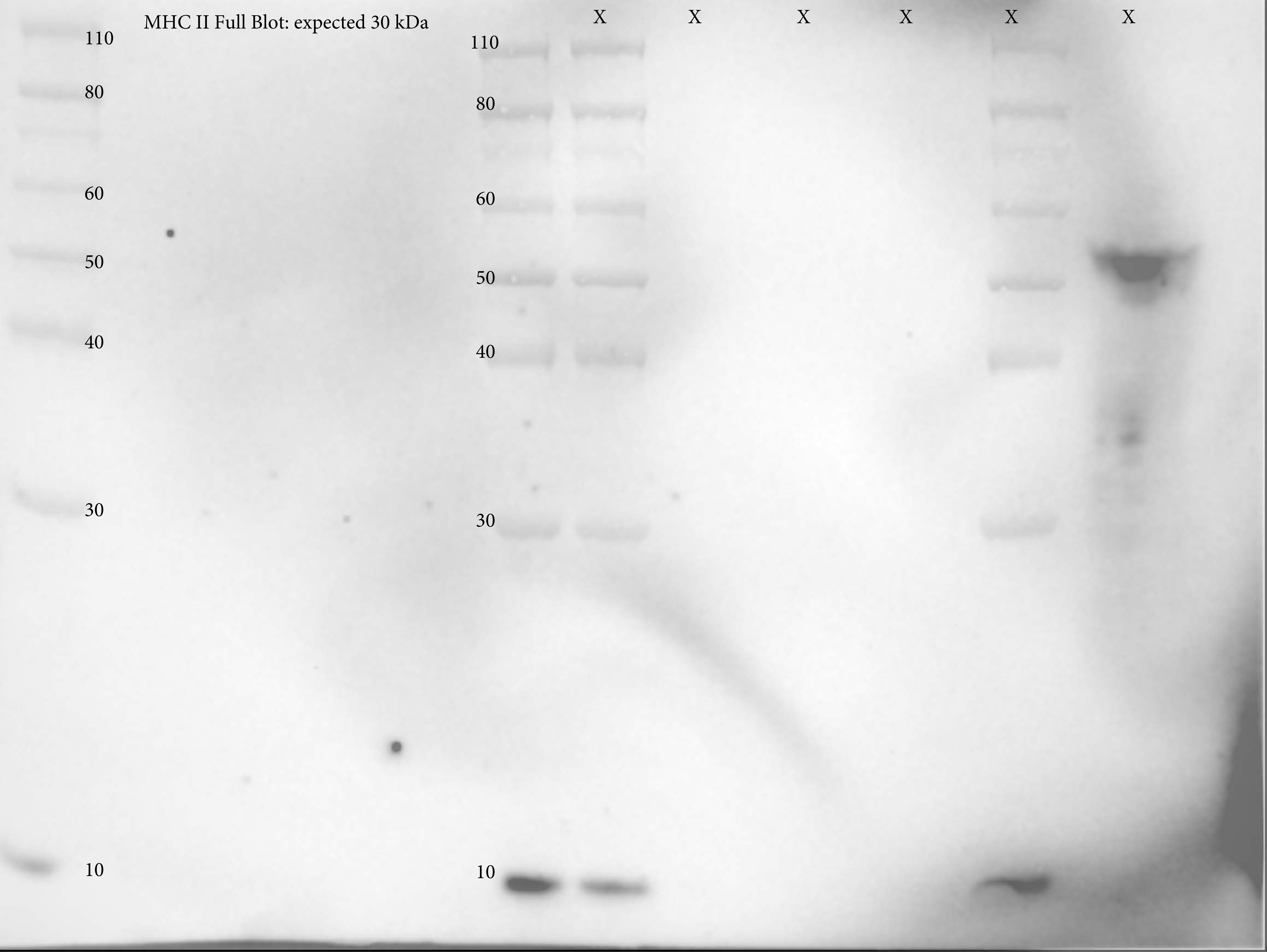

MHC II Full Blot: expected 30 kDa
110
80
60
50
40
30
10
X
X
X
X
X
X
110
80
60
50
40
30
10

MHC I Full Blot: expected 40 kDa

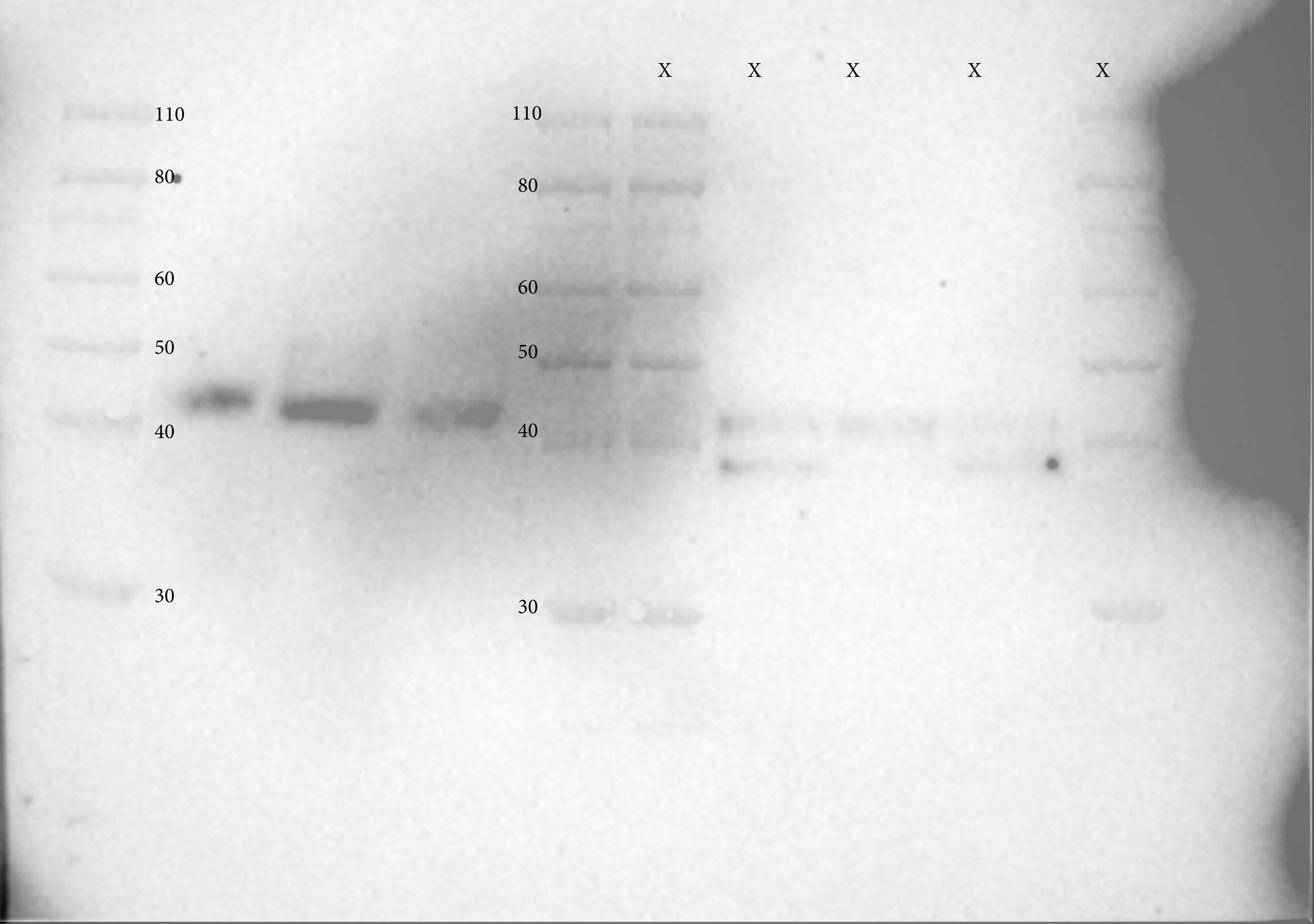

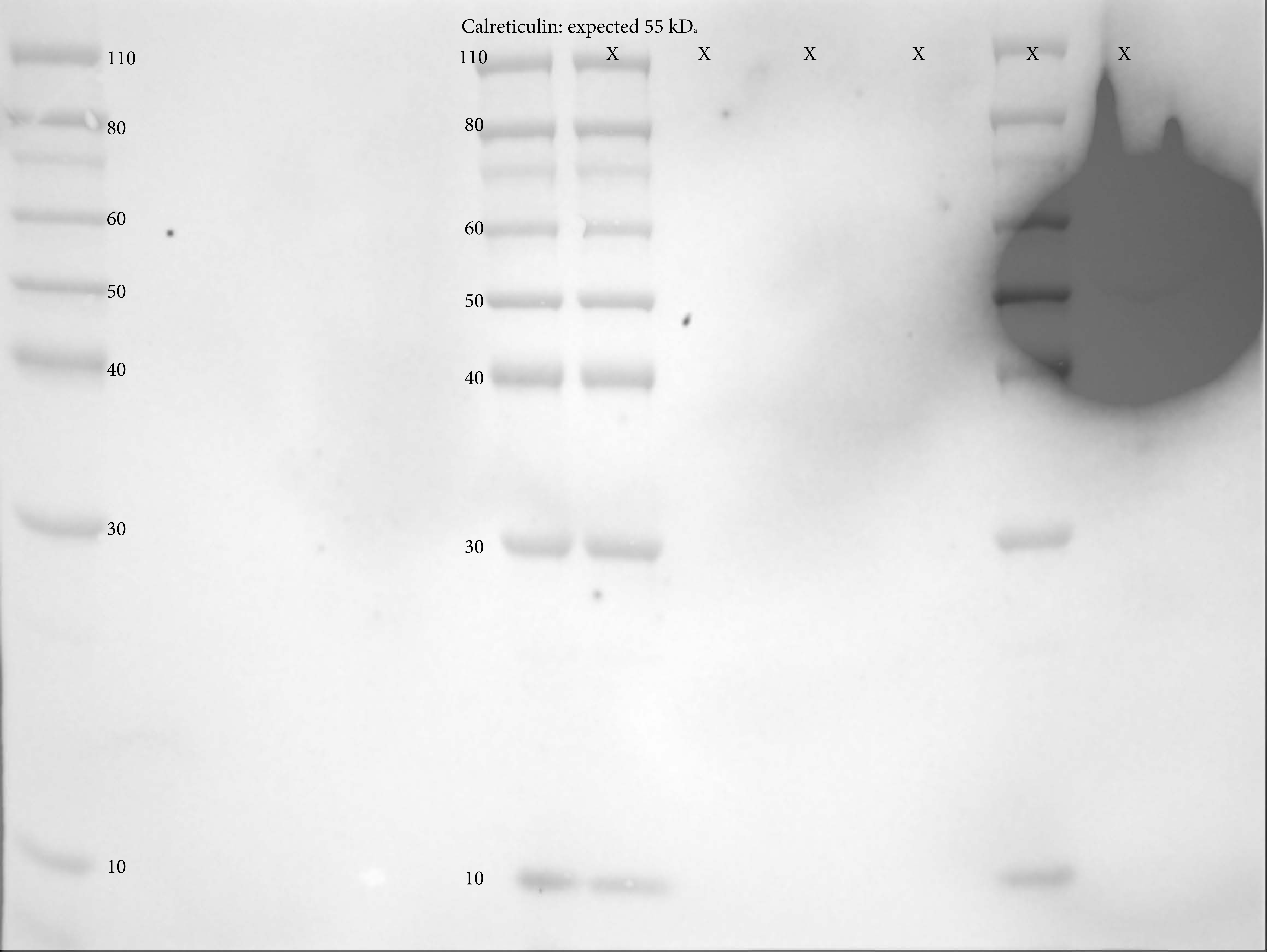
Calreticulin: expected 55 kDa
110
80
60
50
40
30
10
110
80
60
50
40
30
10
X
X
X
X
X
X
